# Unravelling the Intricate Relationship Between Oxidative Stress and Endothelial Dysfunction in Hypertension

**DOI:** 10.7759/cureus.61245

**Published:** 2024-05-28

**Authors:** Ashwani Sharma, Sharanagouda M Patil, Arkajit Dasgupta, Amrit Podder, Jayballabh Kumar, Pooja Sindwani, Priyanka Karumuri

**Affiliations:** 1 Physiology, Teerthanker Mahaveer Medical College & Research Center, Moradabad, IND; 2 Physiology, Shri B M Patil Medical College, Hospital and Research Centre, Vijayapura, IND; 3 Biochemistry, Teerthanker Mahaveer Medical College & Research Center, Moradabad, IND; 4 Preventive Medicine, Teerthanker Mahaveer Medical College & Research Center, Moradabad, IND; 5 Obstetrics and Gynaecology, Apollo CM Fertility, Bengaluru, IND

**Keywords:** endothelial dysfunction, endothelial nitric oxide, hypertension, blood pressure, oxidative stress

## Abstract

Introduction: Hypertension (HTN), a leading risk factor for cardiovascular diseases, is intricately linked with endothelial dysfunction, a hallmark of vascular pathology. The effect of oxidative stress in maintaining the optimum endothelial function in the regulation of blood pressure is yet to be explored. While numerous factors contribute to the pathogenesis of HTN, emerging evidence highlights the pivotal role of oxidative stress in endothelial dysfunction, offering novel insights into the underlying mechanisms.

Aim: Our study delves into the multifaceted relationship between oxidative stress and endothelial dysfunction in HTN, elucidating key molecular pathways and potential therapeutic avenues. Our study aims to find out the association between oxidative stress and endothelial function in the regulation of blood pressure.

Methods: A total of 108 age-matched participants of both genders were divided into three groups by following the guidelines of the American Heart Association (AHA) classification for HTN. Blood pressure was recorded manually in resting posture three times at an interval of 10 minutes using a sphygmomanometer after providing 10 minutes of rest before the first reading. Parameters of oxidative stress and endothelial function were measured by using a UV spectrophotometer. Our study results were depicted as mean ± SD.

Results: The correlation between our variables was performed using Spearman’s correlation considering the value of p<0.05 as statistically significant. Serum malondialdehyde (MDA), a parameter of oxidative stress, was found to be increasing and serum nitric oxide (NO), a parameter to assess endothelial function, was found to be decreasing as the blood pressure increased. These observations are indicative that optimal oxidative stress and optimal endothelial function are required to maintain normal blood pressure regardless of gender.

Conclusions: All persons who are suspected of future cardiovascular risks should be regularly checked for these parameters to avoid cardiovascular morbidity such as HTN.

## Introduction

Hypertension (HTN), characterized by elevated blood pressure (BP) levels, represents a significant global health burden, predisposing individuals to cardiovascular complications such as myocardial infarction, stroke, and heart failure. Endothelial dysfunction, characterized by impaired endothelial function and homeostasis, serves as a critical precursor to vascular dysfunction and contributes to the progression of HTN. Oxidative stress, arising from an imbalance between reactive oxygen species (ROS) production and antioxidant defense mechanisms, has emerged as a central player in the pathogenesis of endothelial dysfunction in HTN [1]. The current scenario after the latest American Heart Association (AHA) guidelines is depicting a worse image of society as most of the current population of hypertensive patients may remain either undiagnosed or don’t seek any medical attention [2]. The effect of oxidative stress and endothelial function in the regulation of BP is yet to be explored. Several studies have shown that hypertensive patients have lower levels of serum nitric oxide (NO) and higher oxidative stress which alter the vascular architecture [3]. Oxidative stress also plays a major role in the promotion of a prothrombic state in vessels whereas endothelial dysfunction is an evident feature of hypertensive vessels [4]. Some studies also showed a higher or non-significant difference in NO level in hypertensive patients as compared to normotensive individuals unless and until there is no associated family history of cardiovascular diseases (CVD) or predisposing cardiovascular risk factors [5,6]. So, this case-control study aims to explore the intricate relationship between oxidative stress and endothelial function in the regulation of BP.

## Materials and methods

Method of collection of data

Our study is a case-control study, performed post-obtaining institutional ethical clearance (IEC/No-09/2021 Dated 22/01/2021) and following voluntary informed written consent from all the study participants [2]. The data for our study were collected between 9:00 AM to 11:00 AM at room temperature for continuous 17 days and analyzed for two days in the laboratory of vascular physiology and medicine of Shri B M Patil Medical College Hospital and Research Centre, BLDE (Deemed to Be University), Vijayapura, Karnataka, India. A total of 108 participants of both genders (54 males and 54 females) were divided into group 1 (control, normotensives, n = 36), group 2 (stage I hypertensives, n = 36) and group 3 (stage II hypertensives, n = 36) from the age range of 35 to 50 years of Vijayapura City, Karnataka, India. Chronic smokers, alcoholics, patients suffering from diabetes mellitus, thyroid diseases or any other chronic illness along with the patients receiving antihypertensive treatments were excluded from our present study. Each participant group of 36 participants contained 18 male participants and 18 female participants.

BP phenotypes measurement

Systolic blood pressure (SBP, mmHg) and diastolic blood pressure (DBP, mmHg) were recorded using a mercury sphygmomanometer after providing 10 minutes of rest before the first measurement. The pulse pressure (PP, mmHg) and mean arterial pressure (MAP, mmHg) were calculated. All the parameters were recorded three times for each of the participants keeping 10 minutes between the recordings of each parameter and the mean value was considered as the final value [2].

Assessment of markers of oxidative stress (serum malondialdehyde; MDA) and endothelial function (serum NO)

After the recording of BP was completed, the fasting blood sample was taken for each of the participants in plain vial and centrifuged at 3500 rpm for 10 minutes following which serum samples were collected and stored at -21 degrees Celsius. The serum samples of the first 10 participants which were collected on the first day were stored for 16 days and on the last day serum samples for seven participants were stored for two days. On the 19th day, all the samples were kept at room temperature and assessed for serum MDA and serum NO by using a UV spectrophotometer at 535 nm [7].

Statistical analysis

The obtained data was entered into an MS Excel sheet (Microsoft® Corp., Redmond, WA, USA). Statistical analysis was performed using Statistical Package for the Social Sciences (IBM SPSS Statistics for Windows, IBM Corp., Version 20.0, Armonk, NY). Data was presented as mean ± SD and diagrams. Categorical variables were compared by using the Chi-square test. Differences between groups of continuous variables were compared using the Mann-Whitney U test, analysis of variance (ANOVA) test, and Kruskal-Wallis test. Spearman’s correlation was used to find a correlation between the variables of physiological parameters, parameters of oxidative stress and endothelial function. p<0.05 was considered statistically significant. All the statistical tests are performed in two-tailed.

## Results

Table 1 shows the comparison of age between different groups of the study (n = 108) which is statistically insignificant (p >0.05) by ANOVA test. As all the samples from the control, stage I HTN and stage II HTN groups showed no significant difference between each other; hence, in this study, all the subjects were found age-matched.

**Table 1:** Comparison of age between three groups Data is represented in the form of mean ± SD. p ≤ 0.05 is taken as statistically significant. ANOVA: analysis of variance; HTN: hypertension

	Control Group (n=36)	Stage I HTN (n=36)	Stage II HTN (n=36)	ANOVA	p-value
Age (Year)	42.75 ± 5.65	43.11 ± 5.73	44.72 ± 4.79	F=1.35	P=0.26

BP phenotypes

While analyzing the BP phenotypes between three groups by using the Kruskal-Wallis test, it showed significantly higher values of BP phenotypes in the stage II HTN group participants as compared to stage I HTN group participants and the same trend was also observed while comparing the stage I HTN group participants with control group participants which is depicted in Table 2. This indicates that the sample selection was following the appropriate methodology for inclusion criteria. Further, results also indicate that there is no gender bias on physiological parameters among the study groups as we did not find any significant difference in any of the physiological parameters between the genders by using the Mann-Whitney U test as depicted in Table 3.

**Table 2:** Comparison of blood pressure phenotypes between three groups Data is represented in the form of mean ± SD. p ≤ 0.05 is taken as statistically significant. SBP: systolic blood pressure; DBP: diastolic blood pressure; PP: pulse pressure; MAP: mean arterial pressure; HTN: hypertension; KW: Kruskal-Wallis

	Control Group (n=36)	Stage I HTN (n=36)	Stage II HTN (n=36)	KW	p-value
SBP (mmHg)	115.1 ± 3.39	134.2 ± 2.76	148.1 ± 8.34	90.197	<0.001
DBP (mmHg)	73.28 ± 4.76	83.17 ± 3.62	90.33 ± 6.07	79.440	<0.001
PP (mmHg)	41.83 ± 5.05	51.00 ± 4.62	57.72 ± 10.3	51.850	<0.001
MAP (mmHg)	87.22 ± 3.65	100.2 ± 2.68	109.7 ± 4.91	92.191	<0.001

**Table 3:** Comparison of blood pressure phenotypes between genders Data is represented in the form of mean ± SD. p ≤ 0.05 is taken as statistically significant. SBP: systolic blood pressure; DBP: diastolic blood pressure; PP: pulse pressure; MAP: mean arterial pressure

Gender	Male (n=54)	Female (n=54)	Mann-Whitney U Test	p-value
SBP (mmHg)	133.3 ± 15.68	131.6 ± 13.53	U=1394.500	0.696
DBP (mmHg)	82.59 ± 9.410	81.93 ± 7.672	U=1382.000	0.639
PP (mmHg)	50.67 ± 9.292	49.70 ± 10.05	U=1344.000	0.482
MAP (mmHg)	99.54 ± 11.09	98.54 ± 8.855	U=1384.500	0.651

Parameters of oxidative stress (serum MDA) and endothelial function (serum NO)

We also found that the oxidative stress parameter like concentration of serum MDA is significantly higher in stage II HTN group participants as compared to stage I HTN group participants and both the HTN group participants had significantly higher serum MDA concentration as compared to control group participants as shown in Table 4. The concentration of serum NO level, which is a marker for endothelial function, was found to be significantly lower in stage II HTN group participants as compared to stage I HTN group participants and both HTN group participants had significantly lower levels of serum NO concentration as compared to control group participants as depicted in Table 4. No significant differences in all these parameters were seen between the genders of each group as shown in Table 5.

**Table 4:** Comparison of parameters of oxidative stress (serum MDA) and endothelial function (serum NO) between three groups Data is represented in the form of mean ± SD. p ≤ 0.05 is taken as statistically significant. MDA: malondialdehyde; NO: nitric oxide; HTN: hypertension; KW: Kruskal-Wallis

	Control Group (n=36)	Stage I HTN (n=36)	Stage II HTN (n=36)	KW	p-value
MDA (μmol/L)	1.014 ± 0.2	1.222 ± 0.4	2.083 ± 0.55	53.09	<0.001
NO (μmol/L)	8.532 ± 1.5	5.694 ± 1.4	3.694 ± 1.16	75.08	<0.001

**Table 5:** Comparison of parameters of oxidative stress (serum MDA) and endothelial function (serum NO) between genders Data is represented in the form of mean ± SD. p ≤ 0.05 is taken as statistically significant. MDA: malondialdehyde; NO: nitric oxide

Gender	Male (n=54)	Female (n=54)	Mann-Whitney U Test	p-value
serum MDA (μmol/L)	1.396 ± 0.51	1.484 ± 0.72	U=1448.500	0.952
serum NO (μmol/L)	5.829 ± 2.27	6.119 ± 2.55	U=1351.500	0.510

Correlation between the parameter of oxidative stress (serum MDA), endothelial function (serum NO) and MAP in the total study population

Table 6 depicts the representation of the correlation between MAP and serum NO and results indicate a positive correlation (r = 0.623) whereas we found a negative correlation between serum MDA and MAP (r = -0.759).

**Table 6:** Correlation between the parameter of oxidative stress (serum MDA), endothelial function (serum NO) and mean arterial pressure (MAP) in the total study population (n = 108). *p ≤ 0.05 is considered as statistically significant. MDA: malondialdehyde; NO: nitric oxide

	Serum MDA (μmol/L)	Serum NO (μmol/L)
MAP (mmHg)	r = 0.623 (p <0.001)*	r = -0.759 (p <0.001)*

## Discussion

Role of oxidative stress in endothelial dysfunction

Endothelial cells, lining the inner surface of blood vessels, play a pivotal role in regulating vascular tone, inflammation, and thrombosis. Under physiological conditions, endothelial-derived NO exerts vasodilatory effects and inhibits platelet aggregation and leukocyte adhesion. However, in HTN, increased oxidative stress disrupts endothelial NO bioavailability through various mechanisms, including scavenging of NO by ROS, decreased endothelial NO synthase (eNOS) activity, and impaired NO signalling pathways. Consequently, diminished NO-mediated vasodilation and enhanced vasoconstriction contribute to elevated BP levels and endothelial dysfunction [6].

Comparison of parameters of oxidative stress (serum MDA) and endothelial function (serum NO) between genders

Oxidative stress parameters in the case of stage II and stage I hypertensive patients were found to be remarkably altered in our findings. Increased MDA in both stage I and stage II HTN indicate altered vascular pathophysiology [7]. Excessive MDA in stage II and stage I HTN in our study may be due to the generation of more ROS, which is a key factor of HTN pathology by modulating the vasomotor system and developing vasoconstriction through angiotensin II. Lower NO levels in stage I and stage II HTN patients indicate lesser bioavailability of NO, which is a potent vasodilator and extremely dependent on the redox signalling system. Increased levels of ROS in our study probably induced vascular remodelling via oxidative damage. Hence, both MDA and NO results in our study confirm the alteration of arterial smooth muscle cells and endothelial cells. The results of NO are also to be considered as a degree of HTN, possibly antioxidant status might have changed simultaneously during HTN which we could not assess and we consider it to be our limitation. Results also indicate that there is no gender bias on parameters of oxidative stress and endothelial function among the study groups [8].

Molecular mechanisms

Oxidative stress-mediated endothelial dysfunction in HTN involves intricate molecular pathways. Excessive ROS production, derived from various sources such as nicotinamide adenine dinucleotide phosphate (NADPH) oxidase, xanthine oxidase, and mitochondria, promotes endothelial activation and inflammation by upregulating adhesion molecules, cytokines, and chemokines. Furthermore, oxidative stress induces endothelial cell apoptosis and senescence, impairing endothelial repair mechanisms and exacerbating vascular injury. Moreover, ROS-mediated oxidative modification of lipids, proteins, and DNA contributes to endothelial dysfunction by disrupting cellular signalling pathways and promoting a pro-inflammatory and pro-thrombotic vascular milieu [7].

Therapeutic implications

Understanding the intricate interplay between oxidative stress and endothelial dysfunction has significant therapeutic implications for HTN management. Antioxidant therapies, targeting ROS generation or enhancing antioxidant defence mechanisms, hold promise in ameliorating endothelial dysfunction and attenuating HTN-related cardiovascular complications. Additionally, lifestyle modifications, including dietary interventions rich in antioxidants, regular physical activity, and stress reduction strategies, may mitigate oxidative stress and preserve endothelial function in hypertensive individuals. Moreover, novel therapeutic approaches targeting specific molecular pathways implicated in oxidative stress-mediated endothelial dysfunction are under investigation and may offer future therapeutic avenues for hypertensive management [8].

## Conclusions

Our study has been undertaken to find the association between oxidative stress and endothelial function in BP. We found that oxidative stress and endothelial function are altered in hypertensive individuals as compared to normotensives. We also found that oxidative stress and endothelial function share a very intricate relationship with BP. Oxidative stress represents a key determinant of endothelial dysfunction in HTN, driving vascular pathology and contributing to cardiovascular morbidity and mortality. Elucidating the intricate molecular mechanisms underlying oxidative stress-mediated endothelial dysfunction provides valuable insights into the pathogenesis of HTN and unveils novel therapeutic targets for CVD management. Hence, we conclude that optimal oxidative stress and endothelial function are required for the maintenance of normal BP regardless of gender. So, serum MDA and serum NO should be checked in routine clinical investigations in all persons who are suspected of future cardiovascular risks such as HTN. Integrating antioxidant strategies with conventional antihypertensive therapies may herald a paradigm shift in HTN management, emphasizing the importance of preserving endothelial function in mitigating cardiovascular risk.

## References

[1] Loffredo L, Violi F (2022). Metabolic endotoxemia and arterial stiffness in diabetes. Circ Res.

[2] Podder A, Patil SM, Kanthe PS (2023). Physical anthropometry influences arterial stiffness in hypertensive patients of North Karnataka. Biomed Pharmacol J.

[3] Armas-Padilla MC, Armas-Hernández MJ, Sosa-Canache B (2007). Nitric oxide and malondialdehyde in human hypertension. Am J Ther.

[4] Ferroni P, Basili S, Paoletti V, Davì G (2006). Endothelial dysfunction and oxidative stress in arterial hypertension. Nutr Metab Cardiovasc Dis.

[5] Higashino H, Miya H, Mukai H, Miya Y (2007). Serum nitric oxide metabolite (NO(x)) levels in hypertensive patients at rest: a comparison of age, gender, blood pressure and complications using normotensive controls. Clin Exp Pharmacol Physiol.

[6] Goch A, Banach M, Mikhailidis DP, Rysz J, Goch JH (2009). Endothelial dysfunction in patients with noncomplicated and complicated hypertension. Clin Exp Hypertens.

[7] Das KK (2022). Vascular physiology: a bridge between health and disease. Indian J Physiol Pharmacol.

[8] Rodrigo R, González J, Paoletto F (2011). The role of oxidative stress in the pathophysiology of hypertension. Hypertens Res.

